# Being safe, feeling safe, and stigmatizing attitude among primary health care staff in providing multidrug-resistant tuberculosis care in Bantul District, Yogyakarta Province, Indonesia

**DOI:** 10.1186/s12960-019-0354-8

**Published:** 2019-03-04

**Authors:** Ari Probandari, Hary Sanjoto, Melani Ratih Mahanani, Luthfi Azizatunnisa, Sampir Widayati

**Affiliations:** 1 Department of Public Health, Faculty of Medicine, Universitas Sebelas Maret, Jalan Ir. Sutami 36A, Surakarta, 57126 Indonesia; 2 Disease Control Research Group, Faculty of Medicine, Universitas Sebelas Maret, Jalan Ir. Sutami 36A, Surakarta, 57126 Indonesia; 3 Bantul District Health Office, Komplek II Kantor Pemerintah Kabupaten Bantul Jalan Lingkar Timur, Manding, Trirenggo, Bantul, Yogyakarta Province 55714 Indonesia

**Keywords:** Safety, Stigma, Health workers, Tuberculosis, Multidrug-resistant tuberculosis

## Abstract

**Introduction:**

Patient-centered care approach in multidrug-resistant tuberculosis care requires health worker safety that covers both being safe and feeling safe to conduct the services. Stigma has been argued as a barrier to patient-centered care. However, there has been relatively little research addressing the issues of safety and stigma among health staff. This paper explored the issue of being safe, feeling safe, and stigmatizing attitude among health staff working with multidrug-resistant tuberculosis cases in primary health care facilities in Indonesia.

**Methods:**

Using a mixed methods research design, data was collected with structured questionnaires among 123 staff, observations of infection control in 17 primary health care facilities, and in-depth interviews among 22 staff.

**Results:**

The findings showed suboptimal infection control infrastructures for the primary health care facilities. The knowledge and motivation to follow multidrug-resistant tuberculosis care protocols are suboptimal. Feeling unsafe is related to stigmatizing attitude in providing multidrug-resistant tuberculosis care.

**Conclusion:**

Being safe, feeling unsafe, and stigmatizing attitude are challenges in providing patient-centered multidrug-resistant tuberculosis care in primary health care facilities in Indonesia. Serious efforts are needed on all levels to ensure safety and prevent irrational stigma.

## Introduction

A patient-centered care approach has been considered as an essential pillar for tackling tuberculosis (TB) global epidemic [1]. Improving a patient-centered care approach, however, cannot be implemented effectively without simultaneously managing the safety of health workers, and it has been recognized that the safety of health workers is an imperative that should receive more serious attention [2].

Health worker safety, however, is not just a matter of being safe. Health workers must also feel safe. In the absence of this broader health worker safety, there is a danger that patient-centered care will be jeopardized because of stigmatizing attitudes toward TB patients by unsafe health workers. Indeed, there is a significant body of literature looking at stigmatizing attitudes among health staff working in TB care, and the adverse consequences on care [3–5].

Indonesia is one of 27 countries with a high burden of multidrug-resistant tuberculosis (MDR-TB) globally [6]. Notwithstanding the burden of MDR-TB in Indonesia and the known impact of stigma on the provision of patient-centered care, there has been a limited number of research addressing the issue. This is particularly unfortunate because it is well known that levels of stigma, as a social construct, vary with context [5, 7].

The Indonesian Ministry of Health has promoted direct observed treatment (DOT) [8, 9] as the strategy in the management of TB. Since 2014, there has been a concerted effort to identify the primary health care facility (PHC) closest to MDR-TB patient’s address as the center of care. A primary health care facility should provide treatment management for MDR-TB patients at the continuation phase.

Bantul district, a district in Yogyakarta Province of Indonesia, adopted the policy of DOT for MDR-TB at PHC settings in 2014. However, in 2016, there was the rejection of staff at PHCs to conduct DOT service for MDR-TB. This paper explored the issue of safety, feeling safe, and stigmatizing attitude among health staff working with MDR-TB cases in PHCs in Bantul district, Yogyakarta Province, Indonesia.

## Methods

### Research settings

Bantul district is one of five districts in Yogyakarta Province of Indonesia, with 971,000 inhabitants. The population density is nearly 2000 per square kilometer. There are 27 PHCs located in 17 sub-districts of Bantul [10]. Seven of 27 PHCs have implemented DOT. The number of presumptive MDR-TB cases has increased in the last 3 years, i.e., 31 cases in 2013, 41 cases in 2014, and 45 cases in 2015 (District Health Office of Bantul, unpublished data).

### Study design

We applied a concurrent nested mixed methods design [11] that combined a health care facility-based cross-sectional study and a qualitative study. The cross-sectional study was conducted to assess the health staff’s knowledge about infection control and their attitude to conduct proper care protocols for MDR-TB. Through the survey, we would like to describe whether health staff’s knowledge about infection control and the attitude to conduct DOT for MDR-TB were lacking. The cross-sectional study is nested in the qualitative observations of infection control infrastructure and staff behavior. The observation aimed to assess the infrastructure and activities related to infection control. We also conducted in-depth interviews to explain the findings from the cross-sectional study and observation, and to explore about reasons behind irrational stigma behavior.

### Research participants and sampling

For the survey, we selected staff that met the inclusion criteria in all 27 PHCs: health staff involved in implementing protocols for MDR-TB care (registration staff, medical doctors, nurses, lab staff) and infection control (staff responsible for health promotion and environmental health). Sample size calculation was done based on the formula for a descriptive cross-sectional study [12]. By estimating that 50% of staff among total 180 population of relevant health staff at PHCs (Bantul district health office 2015, unpublished data) were knowledgeable about the MDR-TB care procedure and infection control, 95% confidence level, and precision 10%, we calculated a minimum sample size of 123 individuals for our study. Data on eligible staff were obtained from the District Health Office. We selected the staff by quota sampling until we reached the minimum sample size.

For the in-depth interviews, we selected health staff at PHCs relevant to MDR-TB care by purposive sampling (medical doctor, nurses, lab staff, and TB program staff) for in-depth interviews. We also conducted observations of infection control facilities with a TB infection control consultant to assess the adequacy of infrastructure and procedures of MDR-TB care in PHCs. Both in-depth interviews and observations were conducted until data saturation was achieved.

### Data collection

Two trained surveyors collected data from surveys among PHC staff under the supervision of the last author. The first and second authors mainly conducted both in-depth interviews and observations of staff behavior in conducting DOT for MDR-TB patients. An infection control expert conducted the observation of infection control infrastructure and proper MDR-TB protocols.

Structured questionnaires about knowledge on infection control and observation guidelines were developed based on the national guideline for infection control related to TB control programs at the health care facility level [13]. The questionnaires and the observation guidelines were piloted and validated. The content validity of the quantitative questionnaires and guideline of in-depth interviews were assessed through the panel of experts during the proposal finalization workshop. We piloted the quantitative questionnaires to 15 primary health care staff to assess their clarity to each question. The observation checklist of infection control was validated by the expert from Universitas Gadjah Mada, Indonesia.

### Data analysis

Qualitative data were analyzed using the content analysis technique [14, 15]. From the text of transcripts, we identified phrases/sentences containing specific meanings (meaning units). The manifest meanings and latent meanings were generated from the meaning units, which were assigned as codes. The codes with similarity were grouped into categories. Open Code software [16] was used for facilitating the data analysis. To improve the trustworthiness of the qualitative data in the data analysis, we conducted peer-debriefing in the researcher team.

Quantitative data were used for triangulation of the qualitative data in the mixed methods design. Quantitative data were analyzed by comparing the knowledge and attitude of staff by their characteristics.

### Research ethics

Ethical approval was obtained from the Ethics Committee, Faculty of Medicine, Universitas Sebelas Maret at Surakarta Indonesia. We obtained written informed consent from all study participants. The researchers conducted the informed consent for the qualitative data collection, while two enumerators conducted the informed consent for the quantitative data collection. The enumerator received training from co-PIs to conduct the informed consent process and collecting data.

## Results

### Characteristics of study participants

A total of 22 in-depth interviews were conducted in 17 PHCs, with 19 in-depth interviews with clinical staff (7 physicians, 1 dentist, 9 nurses, and 2 lab staff) in 17 PHCs, and key informant interviews among three TB program managers at the district/provincial level. Ten out of 19 clinical staff were also acted as TB program staff.

A total of 123 health staff in all 27 PHCs were included in the cross-sectional study (Table 1). The majority of the study informants were clinical staff (medical doctors, nurses, lab staff). On average, the study informants were in the productive age (39 ± 12 years old). Most of them were female (68%), with the level of education of 3 years Diploma. About 90% of health staff has been working for more than 5 years.

**Table 1:** Characteristics of primary health care staff participating for the cross-sectional study

Characteristics	All (*N* = 123)	Clinical staff^a^ (*N* = 95)	Tuberculosis program staff (*N* = 10)	Other^b^ staff (*N* = 18)
Age (years), mean ± SD	39 ± 12	38 ± 12	35 ± 14	47 ± 8
Gender, *n* (%)				
Male	39 (31.7)	27 (28.4)	0 (0)	12 (66.7)
Female	84 (68.3)	68 (71.6)	10 (100)	6 (33.3)
Level of education, *n* (%)				
Diploma	68 (55.3)	48 (50.5)	9 (90.0)	11 (61.1)
Undergraduate	46 (37.4)	39 (41.1)	1 (10.0)	6 (33.3)
Graduate	2 (1.6)	1 (1.1)	0 (0)	1 (5.6)
Professional	7 (5.7)	7 (7.4)	0 (0)	0 (0)
Length of work, *n* (%)				
< 1 year	4 (3.3)	3 (3.2)	0 (0)	1 (5.6)
1–5 years	5 (4.1)	5 (5.3)	0 (0)	0 (0)
6–10 years	33 (26.8)	27 (28.4)	5 (50.0)	1 (5.6)
> 10 years	81 (65.9)	60 (63.2)	5 (50.0)	16 (88.9)
Working at PHCs provides DOT for MDR-TB, *n* (%)	39 (31.7)	30 (31.6)	4 (40.0)	5 (27.8)
Received on-the-job training on DOT for MDR-TB procedure, *n* (%)	36 (29.3)	27 (28.4)	6 (60.0)	3 (16.7)
Received training on infection control, *n* (%)	75 (61.0)	61 (64.2)	8 (80.0)	6 (33.3)

^a^Clinical staff: medical doctor, nurse, and laboratory staff. ^b^Other staff: registration staff, health promotion staff, and environmental health staff

*PHCs* primary health care facilities, *DOT* direct observed treatment, *MDR-TB* multidrug-resistant tuberculosis

### Safety

The observation in 17 PHCs showed suboptimal infrastructure and fidelity of activities to infection control protocols as per the national guideline (Table 2). Proper layout and zoning were only available in eight out of the 17 PHCs; unfortunately, only one PHC provided DOT for MDR-TB had optimal zoning. In addition to zoning, the suboptimal infrastructure of infection control was shown for other types of infrastructures including exhaust fan, infectious disposal system, and sputum collection area. On the other hand, the availability of infectious waste disposal and surgical masks for patients with a cough were adequate.

**Table 2:** Distribution of primary health care facilities with optimal infrastructure and activities of infection control

Domains	Among all observed PHCs (*N* = 17)	Among observed PHCs which provide DOT for MDR-TB (*N* = 7)
	*n*	*n*
Optimal infrastructure		
Layout and zoning	8	1
Airflow	9	2
Available exhaust fan	2	1
Maintain exhaust fan	0	0
Available specific infectious waste disposal	16	7
Infectious waste disposal system	6	3
Air ventilation in laboratory	9	4
Specific sputum collection area	6	5
Available hand scrub liquid	10	5
Available and utilized surgical masks for patients	15	6
Optimal activities		
Triage	14	4
Education	8	3
Separation	10	3

*PHCs* primary health care facilities, *DOT* direct observed treatment, *MDR-TB* multidrug-resistant tuberculosis

The observations also revealed unsafe practices such as health staff’s unawareness about the direction of airflow while they were providing DOT. The health staff even stood in the opposite direction of airflow. The observation also found the health staff unknowingly allowed the MDR-TB patients to be without surgery masks when the patients were having cough during the DOT.

The cross-sectional study revealed that the knowledge of health staff about infection control protocols was inadequate in some domains (Table 3). Health staff had relatively sufficient knowledge about the disposal of tissues used to close mouth when coughing (84%). However, their knowledge about other domains of infection control was still suboptimal. Knowledge about timing to provide cough etiquette were higher among clinical and TB program staff compared to other types of staff (registration, environmental health, health promotion staff). The same pattern was found in terms of setting the infrastructure such as a fan for infection control purpose.

**Table 3:** Distribution of primary health care staff with adequate knowledge on infection control

Domains of knowledge	Total (*N* = 123)	Type of staff				Working at PHCs which provide DOT for MDR-TB			Received on-the-job training on DOT for MDR-TB procedure			Received training on infection control		
		Clinical staff^a^ (*N* = 95)	TB program staff (*N* = 10)	Other staff^b^ (*N* = 18)	*p*	Yes (*N* = 39)	No (*N* = 84)	*p*	Yes (*N* = 36)	No (*N* = 87)	*p*	Yes (*N* = 75)	No (*N* = 48)	*p*
	*n* (%)	*n* (%)	*n* (%)	*n* (%)		*n* (%)	*n* (%)		*n* (%)	*n* (%)		*n* (%)	*n* (%)	
Time to provide education of cough etiquette to patients	91 (74.0)	72 (75.8)	9 (90.0)	10 (55.6)	0.99^c^	31 (79.5)	60 (71.4)	0.343	29 (80.6)	62 (71.3)	0.399^d^	57 (76.0)	34 (70.8)	0.524
Cough screening among visitors	47 (38.2)	41 (43.2)	5 (50.0)	1 (5.6)	0.008	11 (28.2)	36 (42.9)	0.120	14 (38.9)	33 (37.9)	0.921	31 (41.3)	16 (33.3)	0.373
Proper setting of fan in outpatient clinic	88 (71.5)	70 (73.7)	9 (90.0)	9 (50.)	0.050	28 (71.8)	60 (71.4)	0.967	29 (80.6)	59 (67.8)	0.154	59 (78.7)	29 (60.4)	0.029
Disposal of tissues used for closing mouth when coughing	105 (84.5)	81 (85.3)	9 (90.0)	15 (83.3)	0.883^c^	32 (82.1)	73 (86.9)	0.664^d^	32 (88.9)	73 (83.9)	0.667^d^	64 (85.3)	41 (85.4)	1.00
Use of respiratory mask	60 (48.8)	48 (50.5)	5 (50.0)	7 (38.9)	0.661	20 (51.3)	40 (47.6)	0.705	18 (50.0)	42 (48.3)	0.862	37 (49.3)	23 (47.9)	0.878
Use surgical masks among patients during transportation	81 (65.9)	58 (61.1)	7 (70.0)	16 (88.9)	0.071	30 (76.9)	51 (60.7)	0.078	28 (77.8)	53 (60.9)	0.073	50 (66.7)	31 (64.6)	0.812

^a^Clinical staff: medical doctor, nurse, and laboratory staff. ^b^Other staff: registration staff, health promotion staff, and environmental health staff. In general, the data analysis used the Pearson chi-square test. Few analyses used the likelihood ratio chi-square test^c^ and chi-square test with continuity correction^d^

*TB* tuberculosis, *PHCs* primary health care facilities, *DOT* direct observed treatment, *MDR-TB* multidrug-resistant tuberculosis

Additionally, the quantitative data also showed a low proportion of staff with adequate knowledge of infection control in two domains: cough screening among visitors (38%) and use of a respiratory mask (48%) (Table 3). Knowledge about cough screening was lower among staff who were not directly involved in the process of DOT for MDR-TB patients (e.g., registration staff, health promotion, or environmental health staff) compared to clinical or TB program staff (*p* value = 0.008). There was no statistically significant difference between staff working at PHCs with DOT for MDR-TB service and those working in PHCs without DOT for MDR-TB service in terms of knowledge of staff about cough (*p* value = 0.120) and the use of a respiratory mask (*p* value = 0.705). We also found a similar pattern when we compared between staff who received training on DOT for MDR-TB and infection control with those who did not.

### Feeling safe

Our quantitative data showed that approximately half of the health staff stated about feeling stressed and fearful in conducting MDR-TB care (Table 4). Staff at a PHC providing DOT for MDR-TB service perceived less burden of workload compared to the ones at a PHC without the DOT service (56% vs. 32%, *p* value = 0.011). However, there was a relatively similar proportion of staff with perceived fear to be infected or feeling afraid to talk with MDR-TB patients by the type of staff, working/not working at PHCs with DOT, and history of trainings on DOT or infection control.

**Table 4:** Distribution of primary health care staff who stated perceived stress and fear to conduct multidrug-resistant tuberculosis care

Domains of attitude	Total (*N* = 123)	Type of staff				Working at PHCs which provide DOT for MDR-TB			Received on-the-job training on DOT for MDR-TB procedure			Received training on infection control		
		Clinical staff^a^ (*N* = 95)	TB program staff (*N* = 10)	Other staff^b^ (*N* = 18)	*p*	Yes (*N* = 39)	No (*N* = 84)	*p*	Yes (*N* = 36)	No (*N* = 87)	*p*	Yes (*N* = 75)	No (*N* = 48)	*p*
	*n* (%)	*n* (%)	*n* (%)	*n* (%)		*n* (%)	*n* (%)		*n* (%)	*n* (%)		*n* (%)	*n* (%)	
Stress														
Taking account of my workload, I am still able to provide DOT for MDR-TB patients.	49 (39.9)	36 (37.9)	5 (50.0)	8 (44.4)	0.691	22 (56.4)	27 (32.1)	0.011	21 (58.3)	28 (32.2)	0.007	32 (42.7)	17 (35.4)	0.423
I feel stressed providing DOT for MDR-TB patients.	65 (52.8)	14 (14.7)	2 (20.0)	5 (27.8)	0.425	4 (10.3)	17 (20.2)	0.171	2 (5.6)	19 (21.8)	0.055^c^	15 (20.0)	6 (12.5)	0.405^c^
Fear														
I am afraid to be infected when I provide DOT for MDR-TB patients.	58 (47.1)	44 (46.3)	5 (50.0)	9 (50.0)	0.943	18 (46.2)	40 (47.6)	0.880	13 (36.1)	45 (51.7)	0.115	35 (46.7)	23 (47.9)	0.892
I am not afraid to talk with MDR-TB patients when I provide DOT.	58 (47.2)	43 (45.3)	5 (50.0)	10 (55.6)	0.712	19 (48.7)	39 (46.4)	0.813	17 (47.2)	41 (47.1)	0.992	39 (52.0)	19 (39.6)	0.178

^a^Clinical staff: medical doctor, nurse, and laboratory staff. ^b^Other staff: registration staff, health promotion staff, and environmental health staff*.* In general, the data analysis used the Pearson chi-square test. Few analyses used the chi-square test with continuity correction^c^

*TB* tuberculosis, *PHCs* primary health care facilities, *DOT* direct observed treatment, *MDR-TB* multidrug-resistant tuberculosis

The findings from the cross-sectional survey also confirmed a lack of confidence and perceived capacity to provide MDR-TB care in the PHCs (Table 5). Only 65% of health staff felt confident in conducting DOT for MDR-TB patients. Less than 60% of health staff reported their understanding of DOT procedures. Similarly, only 53% of total staff stated that infection control is properly managed in the PHCs. Clinical and TB program staff showed lower confidence in the infection control in PHCs compared to other staff (*p* value = 0.020).

**Table 5:** Distribution of primary health care staff who agree about perceived capacity and motivation to conduct direct observed treatment for multidrug-resistant tuberculosis patients

Domains of attitude	Total (*N* = 123)	Type of staff				Working at PHCs which provide DOT for MDR-TB			Received on-the-job training on DOT for MDR-TB procedure			Received training on infection control		
		Clinical staff^a^ (*N* = 95)	TB program staff (*N* = 10)	Other staff^b^ (*N* = 18)	*p*	Yes (*N* = 39)	No (*N* = 84)	*p*	Yes (*N* = 36)	No (*N* = 87)	*p*	Yes (*N* = 75)	No (*N* = 48)	*p*
	*n* (%)	*n* (%)	*n* (%)	*n* (%)		*n* (%)	*n* (%)		*n* (%)	*n* (%)		*n* (%)	*n* (%)	
Perceived capacity														
I am confident conducting DOT for MDR-TB patient.	81 (65.9)	62 (65.3)	9 (90.0)	10 (55.6)	0.178	32 (82.1)	49 (58.3)	0.01	31 (86.1)	50 (57.5)	0.002	52 (69.3)	29 (60.4)	0.309
I understand the DOT procedure for MDR-TB patient.	72 (58.6)	58 (61.1)	7 (70.0)	7 (38.9)	0.161	31 (79.5)	41 (48.8)	0.001	32 (88.9)	40 (46.0)	< 0.001	48 (64.0)	24 (50.0)	0.124
Infection control is properly managed in the PHC.	66 (53.7)	47 (49.5)	4 (40.0)	15 (83.3)	0.020	24 (61.5)	42 (50.0)	0.232	20 (55.6)	46 (52.9)	0.786	36 (48.0)	30 (62.5)	0.116
Motivation														
If there were a choice, I would prefer not to conduct DOT for MDR-TB patients.	33 (26.8)	28 (29.5)	1 (10.0)	9 (22.2)	0.319	9 (23.1)	24 (28.6)	0.522	6 (16.7)	27 (31.0)	0.158	17 (22.7)	16 (33.3)	0.193
I think I should be involved in TB control by doing DOT for MDR-TB patients.	83 (67.5)	60 (63.2)	10 (100)	13 (72.2)	0.055	28 (71.8)	55 (65.5)	0.486	30 (83.3)	53 (60.9)	0.016	57 (76.0)	26 (54.2)	0.012

^a^Clinical staff: medical doctor, nurse, and laboratory staff. ^b^Other staff: registration staff, health promotion staff, and environmental health staff. Data analysis used the Pearson chi-square test

*TB* tuberculosis, *PHCs* primary health care facilities, *DOT* direct observed treatment, *MDR-TB* multidrug-resistant tuberculosis

Our study also showed the lack of staff motivation to conduct MDR-TB care. Only 67% showed their commitment to providing DOT in accordance to proper protocols for MDR-TB patients. Notably, 26% perceived their preference not to provide proper care, when it was considered a choice (Table 5).

The observation showed inefficiency in the use of the infection control infrastructure of infection control in our observations. For illustration, in a DOT room outside the PHC building that had much airflow, there was an ordinary fan and exhaust fan had been set up. Both fans were used when DOT was provided.

The feeling of being unsafe was also reflected by unnecessary practices by health staff such as wearing double hand gloves, waterproofed gown, plastic boots, and double masks. Those practices reflected efforts to have maximum protection. As an illustration, a nurse at the PHC stated, “I am afraid of being infected… [I know that] the PHC staff who conducts the DOT wear excessive personal protective equipment… I am laughing at them, but I am also afraid… An accident can happen. If we get infected, what is the guarantee? Let’s see, some of our friends got infected, what do they get? Even we are blamed, because of the lack of personal protective equipment…” (Health staff 11, a nurse).

Feeling safe or unsafe to conduct DOT for MDR-TB patients was an interaction between four domains: fear, perceived risk, security, and adequate training (Table 6). Fear was perceived as a health staff’s natural expression when taking care of patients with MDR-TB. However, the perceived risk of being infected varied:

**Table 6:** Examples of meaning units, sub-themes and themes from content analysis of in-depth interviews data about the perception of staff in relation to Directly Observed Treatment for Multidrug Resistant Tuberculosis

Meaning unit	Condensed meaning unit Description close to the text	Condensed meaning unit Interpretation of the underlying meaning	Sub-theme	Theme
“Our main challenge is health staff’s fear toward MDR-TB. One of the factors is uneven knowledge distribution among health staffs. The more we know (about MDR-TB), the more we are scared” (Health staff 4)	Health staff’s fear toward MDR-TB	Fear is a natural expression of health staff when they know about MDR-TB	Fear	Feeling safe/unsafe as an interaction between fear, perceived risk and security, as well as level of knowledge.
“Health staffs were scared to get infected. Fear is human, is not it?” (Health staff 1)	Fear is human	Fear is a natural expression		
	Health staff were scared to get infected	–	Perceived risk	
“They (health staffs) were shocked when they found out there was an MDR-TB patient here” (Health staff 16)	The health staff were shocked when they found out there was an MDR-TB patient at the PHC	–		
“Masks have been provided at the registration desk. There were TB patients here, but health staffs did not wear it. Moreover, there will be MDR-TB patient here” (Health staff 13)	The health staff (at the registration desk) did not wear mask, while there will be MDR-TB patient (at the PHC)	Awareness about risk of being infected is low		
“We are at high risk of getting infected, however there is no certainty on our insurance if we are infected” (health staff 9)	No certainty on our insurance if we are infected	Lack security for staff in doing DOT for MDR-TB patients.	Perceived security	
“They are scared to death. There is no insurance or guarantee of safety from the management” (Health staff 5)	There is no insurance or guarantee of safety from the management			
“…Our intention is to help patient, and we have no choice. We are sure we will be safe as long as our infection control is adequate” (Health staff 11)	We will be safe as long as our infection control is adequate	The infection control is perceived inadequate		
“We will not get infected if the Infection Control is adequate” (Health staff 11)	The health staff will not get infected if the infection control is adequate	Infection control application is perceived essential to prevent MDR-TB transmission		
“First of all, they were afraid to get infected and they are questioning whether the Infection Control has been adequate yet” (Health staff 10)	Health staff are questioning whether the infection control has been adequate yet	The infection control is perceived inadequate		
“It will be useless to give training to the health staff. If they are scared, it’s personal…” (Health staff 2)	It will be useless to give training to give training	Current content of training can be not effective to reduce fear	Knowledge	
“When they (health staffs) were surveyed, the result might show low knowledge. It is because uneven knowledge distribution among health staffs” (Health staff 1)	Uneven knowledge distribution among health staff	Need more staff to be trained		
“…Because we have not got the training so our fear beats everything” (Health staff 10)	Health staff have not got the training	Fear is bigger when the staff have not received training		
“Most of health staffs only focus on their job, for instance nurse and nutritionist did not understand about this matter (MDR-TB)” (Health staff 1)	The health staff did not understand about MDR-TB	Information about MDR-TB is still limited among health staff		
“There is indeed fear toward MDR-TB. Health staffs should understand on infection control of MDR-TB. Therefore, dissemination of information is very important”	Dissemination of information (about infection control of MDR-TB) is important	–		
“Overall, PHCs are ready. There are only few health staff who still discriminate MDR-TB patient because of scared” (Health staff 2)	Few health staff discriminate MDR-TB patients because of scared.	Fear is a driver to a stigmatic attitude to MDR-TB patients	Stigmatic attitude	Fear as a driver of stigmatic attitude
			Fear

We are at high risk of getting infected, however, there is no certainty on our insurance if we are infected (Health staff 9, a nurse)

Masks have been provided at the registration desk. There were TB patients here, but health staffs did not wear it. Moreover, there will be an MDR TB patient here (Health staff 13, a nurse who was also a TB program staff)

The awareness about the risk of being infected increased when the PHC received an MDR-TB patient for DOT, as expressed by an informant, “They (health staffs) were shocked when they found out there was an MDR-TB patient here” (Health staff 16, a nurse who was also a TB program staff).

Perceived risk and lacking security among the staff prompted the domination of fear in their response in regard to DOT for MDR-TB patients. While training was considered as an intervention to reduce the fear, infection control capacity in the PHC was essential. Training only was perceived could not overcome fear.

…Our intention is to help patients, and we have no choice. We are sure we will be safe as long as our infection control is adequate. (Health staff 11, a nurse)

We provided training to our staff, but it could not overcome fear. (Health staff 6, a physician)

Information from the in-depth interviews showed that the coverage and quality of training were perceived inadequate by some health staff at PHCs. The survey data showed that only 29% of health staff received the training on DOT for the MDR-TB procedure. Our confirmation with the TB program managers revealed that the training was conducted in a working day at the PHC as a preparation to receive a back referred patient from the MDR-TB center hospital.

### Stigmatic attitude

The stigmatic attitude was also shown in our observation. Some health staff kept unnecessary distances from the patients when they conducted DOT for MDR-TB care. Even a health staff was talking with the patient from a small window while the patient was in the DOT room and also locked the door of the DOT room. The in-depth interview data revealed that fear was a driver to a stigmatic attitude to MDR-TB patients.

## Discussions

Our study highlighted existing issues about being safe, feeling safe, and stigmatic attitudes among health staff in providing MDR-TB care. Some infrastructures of infection control in PHCs are suboptimal. There was also lacking knowledge of infection control protocols. The stigmatic attitudes manifest in various forms: fear of being infected, avoidance to conduct care, and performing unnecessary overprotected practices of infection control. The cause of the stigmatizing attitude was the fear of being infected. The combination of the suboptimal infection control environment, lacking knowledge, and stigma resulted in unsafe and inefficient practice while doing the MDR-TB care, as well as the discrimination of MDR-TB patients.

Our findings on the inadequacy knowledge and practices of TB infection control such as patients’ screening corroborate previous findings by studies in the hospital setting in South Africa [17, 18]. Low level of knowledge was also worse among administrative staff and other staff who do not have direct relevance to MDR-TB care [19]. Other previous studies in India also found problems with the infection control infrastructure related to drug-resistant TB [20, 21].

The fear among health staff due to MDR-TB transmission risk, while they provide care, is the cause of stigmatizing attitude, as was also found in the previous research studies [22–24]. Stigma among health staff is hypothesized to influence the practice of infection control [23]. Our data revealed empirical evidence that the fear even induced the unsafe and inefficient method of infection control by wearing binary masks (respiratory and surgical masks) and hand gloves. Our study also provided empirical evidence about the influence of fear in the communication between provider and patient that was also proposed by others [22].

### Public health implications

The state of being safe is a priority issue that should be addressed by improving the quality of infection control. Our finding showed a need for knowledge and capacity building for infection control. We argue that this intervention will be a foundation to influence the feeling of safety among health workers to conduct MDR-TB care.

Our study added the gap of knowledge about whether the stigma of health staff to conduct TB care is/is not reasonable. The internalized and enacted stigma among health staff is found related to feeling unsafe due to a lacking infection control infrastructure and knowledge. Improved supervision of MDR-TB care should be performed with the continuation of its implementation.

The findings of stigmatic stigma signified the need for improvement of the quality of care provided by health staff at PHCs. Considering that patient-centered care is one of the pillars of the strategy to end TB [1], we agreed with others that the improvement of the quality of MDR-TB care should include empathy and respect for the patients [24].

Interventions to reduce stigma concerning TB among health care providers are limited [25]. Wu et al.’s study put an effort to decrease internalized stigma among health care workers by training to improve knowledge and attitude among health staff; however, it has been shown less effective among health staff conducting DOT [26]. Hence, more research is needed to get pieces of evidence about the effective strategies to reduce stigma among health staff providing MDR-TB care.

### Study limitation and strength

Our study aimed to explore the phenomenon related to the rejection of the staff to conduct MDR-TB direct observed treatment cases. The study design could not assess any causal relationship between issues of safety, feeling safe, and stigmatizing attitude. On the other hand, the findings from the qualitative data could enrich the interpretation of the quantitative data. Therefore, we could have a holistic portrait of the phenomenon.

This study explored the phenomena of safety and stigmatic attitude with qualitative study which was limited in the generalization in other settings. In future, stigma should be measured quantitatively using a locally adapted instrument. The measurement will be useful to support the evidence related to the level of stigma. Since we argue with the urged need to reduce stigma, the quantitative measurement of stigma among health staff will support the evaluation of the effect from the stigma reduction initiative.

Our study added the gap of knowledge about whether the stigma of health staff to conduct TB care is/is not reasonable in the Indonesian context. We found that the internalized and enacted stigma among health staff is found related to feeling unsafe due to a lacking infection control infrastructure and knowledge. Improved supervision of MDR-TB care should be performed with the continuation of its implementation. The findings from our study contributed to current efforts to understand the nature of stigma among health staff in conducting MDR-TB care that is argued as an initial step to address and tacking the implementation issue of DOT for MDR-TB patients [27]. The way forward to patient-centered MDR-TB care would be jeopardized unless stigma among health staff is diminished.

## Conclusion

Our study concluded with a clear demonstration of lacking safety, feeling unsafe, and stigmatic attitude among health staff to conduct patient-centered MDR-TB care in PHCs in Indonesia. Serious efforts are needed on all levels to ensure safety and prevent irrational stigma.

## Abbreviations

DOT: Direct observed treatment
MDR-TB: Multidrug-resistant tuberculosis
PHC: Primary health care facility
TB: Tuberculosis
